# Family Socioeconomic Position at Birth and School Bonding at Age 15: Blacks’ Diminished Returns

**DOI:** 10.3390/bs9030026

**Published:** 2019-03-11

**Authors:** Shervin Assari

**Affiliations:** 1 Department of Family Medicine, Charles R. Drew University of Medicine and Sciences, Los Angeles, CA 90095, USA; assari@umich.edu or shervinassari@cdrewu.edu; Tel.: +1-323-563-4800 or +1-734-232-0445; 2 Center for Research on Ethnicity, Culture and Health, School of Public Health, University of Michigan, Ann Arbor, MI 48109, USA; 3 Department of Psychiatry, University of Michigan, Ann Arbor, MI 48109, USA

**Keywords:** ethnicity, ethnic groups, African Americans, Blacks, socioeconomic status, socioeconomic position, education, school bonding

## Abstract

Life course epidemiological studies have documented the effects of family socioeconomic position (SEP) at birth on youth developmental processes and outcomes decades later. According to the minorities’ diminished returns (MDR) theory, however, family SEP at birth generates smaller returns for Black compared to White families. Using 15 years of follow up data of a national sample of American families, this study investigated racial differences in the effect of family income at birth on subsequent school bonding of the adolescent at age 15. The fragile families and child well-being study (FFCWS) is a 15-year prospective longitudinal study of 495 White and 1436 Black families from the birth of their child. Family SEP (income to needs ratio) at birth was the independent variable. Youth school bonding at age 15 was the main outcome. Linear regressions were applied for data analysis, with race as the focal moderator. In the pooled sample, in addition to each race, higher family SEP at birth was associated with higher school bonding of the youth at age 15. Race altered the effects of family SEP at birth on youth school bonding at age 15, indicating smaller protective effects for Black compared to White youth. Race stratified regressions also showed the effect of family SEP at birth on age 15 school bonding for White youth, but not Black youth. Tangible outcomes that follow economic resources at birth are disproportionately smaller for Black families compared to those for White families. Merely equalizing SEP is not enough for the elimination of racial inequalities in youth outcomes. Policies should reduce societal and structural barriers that commonly cause diminished returns of SEP for Black families. Policy evaluations should aim for most effective policies that have the potential to equalize Blacks’ and Whites’ chances for gaining tangible developmental and health outcomes from identical SEP resources.

## 1. Background

Socioeconomic position (SEP) promotes population well-being, health, and development [1]. Almost all prospective studies have shown that family and individual SEP, such as lack of poverty status, are among the most protective factors against a wide range of undesired outcomes overall [2,3,4,5]. There is a social gradient in desirable well-being and developmental outcomes, meaning that they are more common in the highest SEP sections of the society, and their prevalence gradually declines as SEP declines [6,7,8]. McLoyd and colleagues conceptualized financial stress and low income as the main mechanism that explains poor developmental outcomes among Black children and youth [9,10,11].

While the overall protective effects of SEP on population outcomes are well-established [12,13,14], emerging literature has documented subpopulation differences in the tangible health and developmental outcomes that follow high SEP [15,16,17]. That is, populations vary in their chance to translate their SEP resources to tangible and desirable outcomes [15,18,19]. Among several mechanisms by which SEP affects population is that high SEP individuals experience lower exposure to risk factors and have a higher access to buffers that can protect them when risk occurs [12,13,14]. High SEP, however, however, does not equally protect families from exposures to environmental and psychological risk factors across all demographic and social groups [20,21,22]. For example, high SEP Blacks report poor mental health [23] and discrimination, as their education does not result in high income. As a result, SEP has a differential impact on the wealth, income, living place, life conditions, and purchasing power of subpopulations [24,25,26,27]. Similarly, the very same SEP resources show smaller effects on health behaviors [12,13,14,28,29], such as substance use [30,31], diet [32] and obesity [33], and health outcomes [34,35], for Blacks than Whites.

A wide range of SEP resources generates a larger increase in economic well-being, material resources and assets [36], social networks [37], and human capital for Whites than Blacks. Although upward social mobility is expected to improve health [18,21,22,38,39,40,41], there are higher psychosocial costs of upward social mobility for Blacks [24,42]. Educational attainment may have a smaller effect on behaviors and human capital for Blacks in comparison to Whites [29,43]. As a result, racial disparities remain, despite similarities in the SEP effects [15,44,45,46]. That is, health gains that follow high SEP may be smaller for racial and ethnic minorities, a phenomenon explained by the minorities’ diminished returns (MDR) theory [16,17].

Although, in theory, equal SEP resources are expected to equally promote outcomes across populations [31,43,46,47,48], this is far from reality. In fact, due to societal factors such as racism that differentially provide access to opportunity structure for social groups, the health gain that follows high SEP depends on a wide range of demographic and social factors, such as race and ethnicity [46,47,49,50,51,52,53,54,55]. The magnitude and the mechanism for the effects of SEP indicators vary across populations [31,47,56]. Social context and life circumstances that differ across demographic and racial groups may bound how ESS translates in health [46,50,57,58,59].

With an assumption that one size fits all, most research on the SEP–health link has not explored the potential racial variation in how SEP indicators impact subpopulation health [60,61]. To give an example, education differently changes the purchasing power as well as life conditions for racial groups [29,34,62].

There are major Black–White gaps in the SEP–developmental/health outcomes link [34,48,57,63]. For example, educational attainment, income, employment, and marital status have stronger effects on smoking [30], diet [32], drinking [31], depression [35], suicidality [62], chronic disease [35], and mortality [34,48,57,63] for Whites than Blacks. High SEP may even increase the risk of poor mental health for Blacks [23]. 

To shed more light on the heterogeneity in the association between family SEP at birth and youth developmental outcomes decades later, this study used follow up data of a national sample of urban families to examine whether race alters the effects of family SEP at birth on youth school bonding at age 15. In line with the minorities’ diminished returns (MDR) theory [16,17], we expected smaller benefits of better family SEP at birth for Black than White youth. As family type may be one reason why family SEP generates less impact on the lives of Blacks than Whites, we ran models in the absence and presence of family type as a covariate to test whether racial variation in the effects of family SEP at birth on future youth outcome is due to racial differences in family type.

## 2. Methods

### 2.1. Design and Setting

With a longitudinal design, the current study used data from the Wave I and Wave 6 of the fragile families and child well-being study (FFCWS), an ongoing population-based cohort (1998–current). Detailed methodological and sampling information on the FFCWS study protocol is published elsewhere [64]. 

### 2.2. Ethics

The Institutional Review Board (IRB) of Princeton University approved the study protocol. All youth legal guardians signed the study informed consent. Adolescents provided assent. 

### 2.3. Participants and Sampling

The FFCWS recruited a national and random sample of families from twenty US cities with a population of 200,000 or more. The FFCWS sample is a cohort of parents from the time of child birth. The study recruited approximately 4655 families (2407 Black, 1354 Hispanics, and 894 White). This number included 3600 nonmarital and 1100 marital couples from 75 hospitals in 20 US cities [64,65,66]. As FFCWS oversampled nonmarried families [64,67], it should not be regarded as a representative sample of US couples. Most FFCWS participants were low SEP and were in nonmarital unions. 

### 2.4. Analytical Sample 

The current analysis used baseline (Wave 1) and 15-year follow up data (Wave 6) of 1931 youth being followed from birth to age 15 [White (*n* = 495) and Black (*n* = 1436)].

### 2.5. Measures

*Main Independent Variable.* Family SEP (household income to needs ratio) measured at baseline was treated as a continuous variable. 

*Covariates.* All covariates were measured at baseline (birth) and included the age of the mother, youth gender, maternal education, and family type. The age of the mother was a continuous measure. Youth gender was a dichotomous measure (1 boy, 0 girl). Family type was a dichotomous variable based on family type, reported by the mother (married 1, all other situations 0). Maternal educational attainment was a continuous variable measured with the following levels: Less than high school, high school completed, some college, and college completed/graduate level.

*Dependent Variable.* Youth school bonding at age 15 was measured using the following four items: (1) Feel close to people at school, (2) Feel like part of school, (3) Happy to be at school, and (4) Feel safe at school. These four items measure the degree of inclusiveness, closeness, happiness, and safety that a teen experiences at school. These items were used by the Panel Study of Income Dynamics and National Longitudinal Study of Adolescent Health (Add Health) to measure the school bonding. At Year 15 of the FFCWS, items were modified to four response scales on a scale of 1 = “strongly disagree” to 4 = “strongly agree.”

### 2.6. Missing Data

Attrition was mainly due to gradual drop out of participating families over a long term follow up period (15 years). From 2923 Black and White families who were recruited into the FFCWS at baseline, 1931 families had data on youth school bonding at age 15. Family type and household income at baseline were not associated with attrition; however, race, maternal education, maternal age, and child gender were associated with attrition. Families who were White and had older and low education mothers with boys were at higher risk of attrition. Thus, attrition was not at random and Black families, and families with girls, high education, and low age of mothers were more likely to be followed for the whole 15 years and thus be included in the current analysis. 

### 2.7. Statistical Analysis

To adjust for the FFCWS sampling weights, Stata 15.0 (Stata Corp., College Station, TX, USA) was applied for data analysis purposes. The jackknife method was used to calculate the weight based standard errors. Proportions and means (SEs) were reported for descriptive purposes. We ran eight linear regression models, four of them in the pooled sample and four by race. To test whether family type explains the SEP by race interaction, we ran four models (1a to 4a) without and four models (1b to 4b) with family type as a covariate. In all models, youth school bonding at age 15 was the outcome, family SEP at birth (income to needs ratio) was the predictor, and mother age, youth gender, and maternal education were the covariates. Models 1a and 1b were run in the pooled sample in the absence of the interaction term. Models 2a and 2b were run in the pooled sample in the presence of the interaction term between maternal education and race. Models 3a and 3b were run in Whites and Models 4a and 4b were run in Blacks. Adjusted beta (b), SE, 95% confidence intervals (CIs), t, and *p* values were presented. *p* of 0.05 or less was considered as significant.

## 3. Results

### 3.1. Descriptive Statistics

The study included 1931 families and their youth who could be successfully followed up for 15 years from birth. This number was composed of 495 White and 1436 Black families.

Table 1 summarizes all the study variables at baseline as well as at age 15 in the pooled sample and by race. Family SEP (poverty status) was higher for White than for Black families. Most White mothers were married to their partners; however, most Black mothers were unmarried. White mothers had higher maternal education than Black mothers. Youth school bonding at age 15 was higher for White than Black youth (Table 1).

### 3.2. Models in the Absence of Family Type

Table 2 summarizes the results of two linear regressions in the pooled sample and two for Whites and Blacks. *Model 1a* did not include any interaction between SEP and race. *Model 2a* did include educational attainment and family type. *Model 3a* and *Model 4a* ran the same model for Whites and Blacks, respectively. *Model 1a* showed that race was marginally associated with youth school bonding at age 15, with Black youth having lower school bonding at age 15 than White youth. This model did not show the effect of income to needs ratio on the outcome. *Model 2a* showed race by family SEP interaction, with a smaller effect of family SEP for Blacks compared to Whites. Stratified linear regression models in White and Black families showed that high family SEP is associated with higher school bonding at age 15 for White, but not Black families (Table 2).

### 3.3. Models in the Presence of Family Type

Table 3 summarizes the results of four linear regressions, two for the pooled sample, one for Whites, and one for Blacks. *Model 1b* did not include any interaction between SEP and race. *Model 2b* did include educational attainment and family type. *Model 3a* and *Model 4a* were ran for Whites and Blacks, respectively. *Model 1b* showed that race was marginally associated with youth school bonding at age 15, with Black youth having lower school bonding at age 15 than White youth. This model did not show the effect of income to needs ratio on the outcome. *Model 2b* showed race by family SEP interaction, with smaller effect of family SEP for Blacks compared to Whites. Stratified linear regression models in White and Black families showed that high family SEP was associated with higher youth school bonding at age 15 for White but not Black families (Table 3).

## 4. Discussion

Racial differences were found in the effects of family SEP at birth on school bonding of the youth at age 15. Only White youth with higher family SEP showed more school bonding at age 15, yet this effect was missing for Black youth. Racial differences in family type did not explain racial differences in the gain from family SEP. 

The finding of this study is in line with the minorities’ diminished returns (MDR) theory [15,34,62,68], which suggests tangible outcomes that follow high SEP are smaller for minorities than for Whites. Several studies have documented similar patterns for other domains, SEP indicators, age groups, designs, and outcomes [16,17,69]. The effects of education, income, and family type on smoking [30], diet [32], drinking [29], anxiety [70], depression [35], sleep quality [34], physical activity [34], and obesity [34,71,72] are smaller for Blacks than for Whites.

Our findings do not suggest that racial difference in family type is the reason behind racial heterogeneity in the effects of family income for Whites than for Blacks [33,70]. We found these patterns hold after controlling for family type. This result contributes to the ongoing debate of whether family type explains some of the poor outcomes for Blacks. Political parties should stop blaming the victim, as a lower marriage rate among Blacks is not the main reason why health and economic disparities exist. Even the married family type also generates smaller effects for Whites than for Blacks [33,70].

These results have applications for the conceptualization of race and SEP and may inform future research and theory in this field. While most classic theories, such as the fundamental cause theory (1995), conceptualize SEP as a fundamental cause for undesired outcomes [12,13,14], it should be remembered that SEP does not function similarly for all groups. 

Race [34], other social identities [7,57,73], and their intersections [38,42,43] alter the outcomes of high SEP [29,74,75,76,77,78,79,80]. Systemically, tangible gains that follow access to SEP resources are smaller for Blacks [74] and Hispanics than for Whites. At least for Blacks, not only are there diminished returns of SEP, but some reports suggest that high SEP increases risk of undesired mental health outcomes [35,62]. High SEP Caribbean Black women report more suicidal ideation than their low SEP counterparts [62] and high SEP Black males are at an increased risk of depressive symptoms [35] and major depressive disorder (MDD) [23].

Several reasons may be involved in such diminished returns of high SEP. Education quality is lower in predominantly Black areas, so the effect of education on life conditions and behaviors would be smaller for them. Due to labor market preferences and practices, as well as residential segregation that shape job availabilities, high SEP Blacks may work at lower earning occupations that are still stressful and do not maximize gains that should follow SEP. As a result, SEP does not give the same boost to life conditions for Blacks compared to Whites [16,17]. Upward social mobility imposes additional psychological and social costs to Blacks [81]. This is particularly due to the unfair treatment by society, which makes it harder for non-Whites to climb the social ladder. To tackle barriers due to multilevel racism and discrimination, Blacks apply effortful behavioral coping, such as John Henryism and experience goal striving stress [82,83,84,85]. These psychologically taxing coping mechanisms reduce health gains that commonly follow the process of upward social mobility for Blacks [86,87]. Thus, upwardly mobile Black individuals and families do not report very a high level of health [19,21,22,88].

## 5. Limitations

The current study had a few limitations. First, although national, sampling was not at random. Thus, selection bias is possible. In addition, due to long follow up period, there was a considerable proportion of attrition, which was not at random. Participants’ characteristics such as race, child gender, and maternal education and age impacted the likelihood of being including in the current analysis. Such selective attrition should be considered when the results are being interpreted. In addition, characteristics of the mother, but not the father were included in this study. Lastly, only individual level SEP was considered. Contextual characteristics, such as neighborhood SEP, may also be important. Despite these limitations, current research makes a unique contribution to the field of race, SEP, and child development.

## 6. Conclusions

To conclude, we found Black–White differences in the effects of family SEP at birth on youth school bonding at age 15, regardless of family type. Racial and ethnic groups unequally gain benefits from their family SEP, meaning Black youth gain less health compared to White youth, not because of racial differences in marital status, but possibly because of societal and structural barriers due to racism. Future research should go beyond behavioral profiles and study particular societal and structural barriers that hinder Black families from translating their SEP resources to tangible and desirable development and health outcomes. There is also a need to find the public and economic policies that can effectively reverse and eventually eliminate racial differences in the actual *effects* of SEP on health (e.g., education, income, and occupation). Policy makers should have in mind that universal increase in SEP may better serve Whites than Blacks and may defeat its purpose, as it may not be a good solution to elimination of racial gaps in health and developmental outcomes.

## Figures and Tables

**Table 1:** Demographic factors in the pooled sample and by race.

	All		Whites		Blacks	
	*n* = 1931		*n* = 495		*n* = 1436	
	%	SE	%	SE	%	SE
Gender						
Female	46.23	0.03	48.92	0.04	42.07	0.04
Male	53.77	0.03	51.08	0.04	57.93	0.04
Married *^,a^						
No	37.69	0.02	18.81	0.02	66.88	0.03
Yes	62.31	0.02	81.19	0.02	33.12	0.03
	Mean	SE	Mean	SE	Mean	SE
Maternal Education *^,b^	2.60	0.04	2.96	0.07	2.04	0.06
Family Income *^,b^	47.86	2.69	64.25	4.45	26.77	2.30
School Bonding *^,b^	13.96	0.16	14.41	0.19	13.40	0.25

* *p* < 0.05 for comparison of Black and White families; ^a^ Pearson Chi square test; ^b^ Independent sample *t* test.

**Table 2:** Summary of linear regression models without family type as a covariate in the pooled sample and by race.

	b(SE)	95% CI	t	*p*
*Model 1a (All)*				
Race (Black)	−0.56(0.32)	−1.21 to 0.09	−1.74	0.091
Youth Gender (Male)	0.28(0.27)	−0.26 to 0.82	1.05	0.302
Maternal Age	−0.02(0.03)	−0.08 to 0.04	−0.69	0.493
Maternal Education	0.42(0.23)	−0.05 to 0.89	1.81	0.080
Income to Needs Ratio	−0.44(0.71)	−1.89 to 1.00	−0.62	0.539
Intercept	13.53(0.67)	12.17 to 14.89	20.31	0.000
*Model 2a (All)*				
Race (Black)	−0.84(0.40)	−1.65 to −0.03	−2.11	0.043
Youth Gender (Male)	0.24(0.28)	−0.33 to 0.82	0.87	0.391
Maternal Age	−0.02(0.03)	−0.08 to 0.03	−0.85	0.401
Maternal Education	0.35(0.21)	−0.09 to 0.79	1.63	0.113
Income to Needs Ratio	−3.47(1.05)	−5.61 to −1.33	−3.30	0.002
Race × Income to Needs Ratio	3.46(1.34)	0.72 to 6.20	2.58	0.015
Intercept	13.97(0.72)	12.51 to 15.43	19.49	0.000
*Model 3a (Whites)*				
Youth Gender (Male)	0.44(0.34)	−0.25 to 1.14	1.30	0.203
Maternal Age	−0.01(0.04)	−0.10 to 0.08	−0.23	0.820
Maternal Education	0.51(0.27)	−0.05 to 1.07	1.84	0.074
Income to Needs Ratio	−2.92(1.07)	−5.10 to −0.73	−2.72	0.011
Intercept	12.93(1.01)	10.86 to 14.99	12.76	0.000
*Model 4a (Blacks)*				
Youth Gender (Male)	−0.07(0.51)	−1.12 to 0.98	−0.14	0.889
Maternal Age	−0.03(0.02)	−0.08 to 0.02	−1.35	0.188
Maternal Education	0.02(0.32)	−0.64 to 0.67	0.05	0.961
Income to Needs Ratio	−0.23(0.77)	−1.79 to 1.34	−0.29	0.770
Intercept	14.29(0.77)	12.73 to 15.85	18.64	0.000

Outcome: Youth school bonding at age 15, Confidence Interval (CI).

**Table 3:** Summary of linear regression models with family type as a covariate in the pooled sample and by race.

	b(SE)	95%CI	t	*p*
*Model 1b(All)*				
Race (Black)	−0.79(0.38)	−1.56 to −0.02	−2.09	0.045
Youth Gender (Male)	0.38(0.24)	−0.12 to 0.88	1.55	0.132
Maternal Age	0.00(0.03)	−0.05 to 0.06	0.14	0.891
Maternal Education	0.57(0.27)	0.02 to 1.13	2.12	0.042
Married	−1.10(0.66)	−2.45 to 0.26	−1.65	0.109
Income to Needs Ratio	−0.51(0.60)	−1.74 to 0.71	−0.85	0.400
Intercept	13.18(0.71)	11.72 to 14.63	18.44	0.000
*Model 2b(All)*				
Race (Black)	−1.04(0.45)	−1.97 to −0.12	−2.30	0.028
Youth Gender (Male)	0.34(0.26)	−0.18 to 0.86	1.33	0.192
Maternal Age	0.00(0.03)	−0.06 to 0.06	−0.05	0.962
Maternal Education	0.50(0.24)	0.02 to 0.98	2.12	0.042
Married	−1.04(0.59)	−2.24 to 0.16	−1.76	0.088
Income to Needs Ratio	−3.37(0.89)	−5.19 to −1.55	−3.78	0.001
Race × Income to Needs Ratio	3.27(1.09)	1.04 to 5.49	2.99	0.005
Intercept	13.61(0.70)	12.19 to 15.03	19.52	0.000
*Model 3b(Whites)*				
Youth Gender (Male)	0.44(0.35)	−0.27 to 1.15	1.26	0.216
Maternal Age	0.00(0.04)	−0.09 to 0.09	0.00	0.998
Maternal Education	0.55(0.32)	−0.11 to 1.21	1.70	0.098
Married	−0.36(0.57)	−1.52 to 0.81	−0.62	0.537
Income to Needs Ratio	−2.89(1.04)	−5.01 to −0.77	−2.77	0.009
Intercept	12.79(1.15)	10.44 to 15.13	11.12	0.000
*Model 4b(Blacks)*				
Youth Gender (Male)	0.26(0.37)	−0.49 to 1.01	0.71	0.485
Maternal Age	0.00(0.02)	−0.05 to 0.04	−0.16	0.873
Maternal Education	0.26(0.31)	−0.38 to 0.90	0.83	0.414
Married	−1.50(0.88)	−3.29 to 0.29	−1.71	0.097
Income to Needs Ratio	−0.34(0.60)	−1.56 to 0.89	−0.56	0.577
Intercept	13.38(0.84)	11.67 to 15.08	15.96	0.000

Outcome: Youth school bonding at age 15, Confidence Interval (CI).
